# Quality assessment of biomass pellets available on the market; example from Poland

**DOI:** 10.1007/s11356-024-33452-1

**Published:** 2024-05-01

**Authors:** Agnieszka Drobniak, Zbigniew Jelonek, Maria Mastalerz, Iwona Jelonek, Kamila Widziewicz-Rzońca

**Affiliations:** 1 Faculty of Natural Sciences, University of Silesia in Katowice, Będzińska 60 St., 41-200 Sosnowiec, Poland; 2 Indiana Geological and Water Survey, Indiana University, 1001 E. 10Th St., Bloomington, IN 47405 USA; 3 Centre for Biomass Energy Research and Education, University of Silesia in Katowice, Będzińska 60 St, 41-200 Sosnowiec, Poland; 4 Institute of Environmental Engineering, Polish Academy of Sciences in Zabrze, M. Skłodowskiej-Curie 34 St., 41-819 Zabrze, Poland

**Keywords:** Biomass pellets, Wood pellets, Quality assessment, Pellet fuel certification, Biomass fuel contaminants, Reflected light microscopy

## Abstract

This study evaluates the quality of 30 biomass pellets sold for residential use in Poland. It provides data on their physical, chemical, and petrographic properties and compares them to existing standards and the information provided by the fuel producers. The results reveal considerable variations in the quality of the pellets and show that some of the purchased samples are not within the DINplus and/or ENplus certification thresholds. Among all 30 purchased samples, only one passes the quality thresholds set by the PL-US BIO, a newly established quality certification in Poland that combines quality assessment following DINplus with optical microscopy analysis. The primary issues causing a decrease in pellet quality include elevated ash and fines content, compromised mechanical durability, too low ash melting temperature, and additions of undesired additions like bark, inorganic matter, and petroleum products. Our research highlights the need for improved fuel quality control measures, and transparent and accurate product labeling, as well as the need for a comprehensive and publicly available national database of solid biomass fuel producers and fuels sold. These are essential steps toward increasing customers’ awareness and trust, encouraging them to embrace biomass fuels as reliable and sustainable sources of energy.

## Introduction

The production and utilization of biomass pellets in Poland have undergone remarkable expansion (Bioenergy Europe 2022, 2023) as a result of political commitments to raise the share of renewable energy in Europe (European Council 2023) and the implementation of a long-term vision of energy transition policy in Poland (PEP2040 2021). The growth is backed by incentives of the Clean Air Program, a Polish government subsidy for the thermo-modernization of residential homes, and the replacement of outdated heating sources (CAP 2024).

**Fig. 1:**
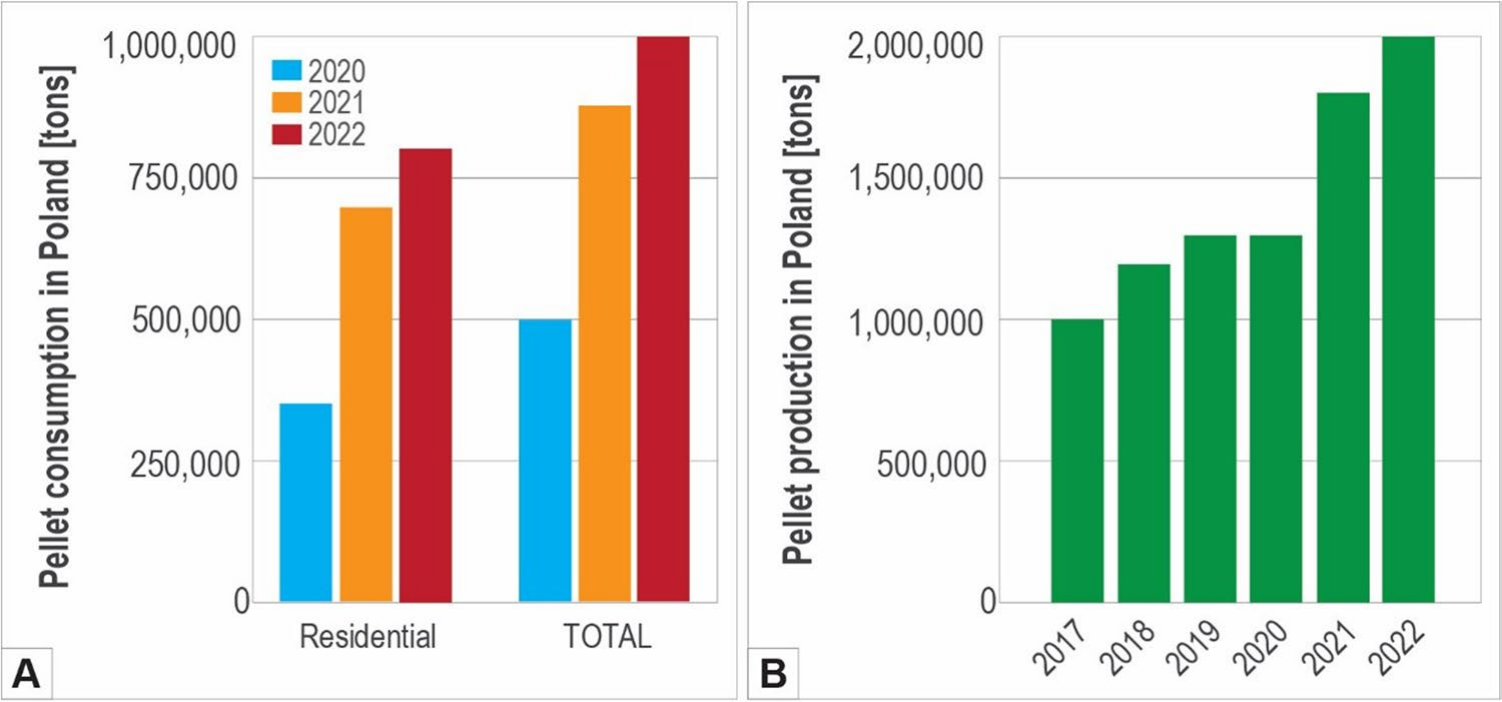
Bar graphs showing the evolution of pellet consumption for heating (**A**) and pellet production in Poland (**B**). Total pellet consumption includes the volume of pellets used for residential, commercial, combined heat and power, and power generation only (Bioenergy Europe 2022, 2023)

Between September 2018, and March 2024, more than 807,000 applications were received to participate in the Clean Air Program with approximately 21% of the applicants opting to switch their home systems to biomass boilers (CAP 2024). Notably, while 8,000 residential wood pellet boilers (< 50 kW) were sold in Poland in 2020, this figure skyrocketed to 42,000 in 2021, and about 40,000 in 2022 (Bioenergy Europe 2022, 2023). Consequently, residential wood pellet consumption for heating surged from 350,000 tons in 2020 to a rapid increase of 700,000 tons in 2021 and 800,000 tons in 2022 (Fig. 1A).

In response to the increased demand for energy in general, the biomass industry in Poland is experiencing dynamic growth (Biomasa 2021, 2023; Bioenergy Europe 2022, 2023; CAP 2024). Pellet production in Poland, estimated at about 1 million tons in 2017, reached 1.8 million tons in 2021 and 2 million tons in 2022 (Fig. 1B), placing Poland as the sixth biggest pellet producer in the world after the USA, Canada, Germany, Vietnam, and France. To allow for such an increase, the number of operating pellet production plants, reported at 55 in 2017, increased to 90 in 2021 and 130 in 2022 (Bioenergy Europe 2019, 2022, 2023).

However, total pellet production within Poland is probably underestimated primarily due to the existence of numerous small-scale pellet producers whose production data are typically unreported. Moreover, in addition to wood pellets, Poland is increasingly utilizing various non-woody biomass materials, including agricultural residues like grass, straw, or sunflower husks, for pellet production. The extent of the production and sales of these types of fuels remains largely unknown, despite their growing popularity.

Because of this increased production, Poland has also emerged as a significant pellet exporter. In 2022, more than 300,000 tons of pellets was exported, mainly to Italy (112,873 tons), Germany (95,755 tons), Denmark (51,513 tons), and France (12,197 tons) (Bioenergy Europe 2022, 2023). With one of the largest biomass potentials in Europe (Jezierska-Thöle et al. 2016; Zyadin et al. 2018), and driven by entrepreneurial initiatives focused on biomass utilization, it is anticipated that the Polish biomass industry will continue to thrive. Given Poland’s ongoing reliance on primary energy sources, investments in renewable energy resources are paramount for the nation’s socio-economic advancement and its alignment with the goals of the European Green Deal (LaFontaine et al. 1989; Baum et al. 2013; Gołuchowska et al. 2015; Piwowar and Dzikuć 2016; Klepacka and Florkowski 2019, 2020; Bełdycka-Bórawska et al. 2021; Pietrzak et al. 2021; Igliński et al. 2022; Wieruszewski et al. 2022b).

Although biomass-based energy is widely recognized as a crucial pathway toward achieving net-zero emissions, the rapid expansion of pellet production in Poland and across the globe has sparked questions and concerns. Studies raised concern about the potential impact of biomass harvest and utilization on human health and the environment, the necessity for responsible and sustainable biomass sourcing, and the urgent need for enhanced forest and biodiversity protection. Recent research and climate activists have also cast doubts on the classification of wood pellets as a renewable energy source and have called for revisions in carbon accounting methodologies for harvested wood and pellet production (Sterman et al. 2018, 2022; Bełdycka-Bórawska et al. 2021; Roszkowska and Szubska-Włodarczyk 2021; Wieruszewski et al. 2022b; Rybarczyk 2023; Shumway 2023). At the same time, to foster the growth of the biomass industry, various challenges like constraining raw materials, improving the efficiency of production, and increasing fuel quality must be addressed. Equally important are socio-economic implications and expanding public awareness about biomass utilization in a safe and environmentally responsible way (Abbasi and Abbasi 2010; Gołuchowska et al. 2015; Piwowar and Dzikuć 2016; Zyadin et al. 2018; Olsztyńska 2019; Klepacka and Florkowski 2019, 2020; Modelska et al. 2020; Senila et al. 2020; Bełdycka-Bórawska et al. 2021; Ślusarz et al. 2021; Zimon et al. 2021; Wieruszewski et al. 2022a, b; Stolarski et al. 2022; Janiszewska and Ossowska 2023; Mawusi et al. 2023).

One of the important aspects of biomass utilization is fuel quality, which is directly connected to the emissions they generate. Given biomass’s growing role as a significant source of renewable energy, recent research highlights the need for a more comprehensive assessment of solid fuel quality (Jelonek et al. 2020, 2021; Drobniak et al. 2021, 2022, 2023) and accurate labeling of pellet products by manufacturers. Such labeling should include information regarding pellet properties and biomass sources (Biomasa 2021).

At present, two leading international wood pellet quality certification programs exist in Poland, ENplus and DINplus, in addition to the DobryPelet certification initiative developed by the Polish Pellet Council and the recently established PL-US BIO program of the Centre for Biomass Energy Research and Education (DINplus 2021; PL-US BIO 2023; PPC 2023; ENplus 2023). Although complete data on the exact number of pellet producers and their production levels in Poland remains undisclosed, data reveal that Poland consistently ranked within the top six ENplus-certified pellet-producing countries between 2015 and 2023. In 2021, approximately 550,000 tons of Polish pellets was certified, and this figure had increased to around 700,000 tons by December, 2023, with a total of 88 active certified producers in March, 2024 (ENplus 2024a, b). Additionally, the DINplus website lists 43 Polish companies as recipients of their certification (DINplus 2023a). However, considering available production data (Bioenergy Europe 2022, 2023) despite the high number of certifications, only about 30% of Polish wood pellets underwent quality assessment and received ENplus certification in 2022, while the amount of certificated pellets by DINplus remains unknown.

It is important to mention that the ENplus fraud management team has observed a significant increase in certificate falsifications over the past 2 years (Bioenergy Europe 2022, 2023). This increase encompasses various deceptive activities, including marketing fraud, fraudulent use of the ENplus trademark on pellet packaging, and the creation of bogus websites that mimic the official online presence of authentic certified companies. This rise in fraudulent practices can be attributed to the growing demand for pellets, which has provided ground for these activities to flourish. This surge in reported fraud cases has been particularly pronounced in Poland, which accounted for 15% of all reported fraud cases in 2022. Moreover, between January and September, 2023, Poland accounted for 20% of the 146 cases reported, becoming the second-highest contributor, after Turkey, of fraudulent cases (Bioenergy Europe 2022, 2023).

It is also essential to highlight the limited accessibility of crucial statistics concerning Poland’s pellet industry. Biomass organization members typically have exclusive access to comprehensive annual reports, and obtaining such access can involve considerable expenses. This limited access to the data presents a notable impediment for individuals who lack the financial means to obtain this information, leading to a lack of transparency in data dissemination. Of paramount concern in this context is the absence of a centralized national database that could provide up-to-date and openly accessible information about the Polish pellet industry. Such a database could serve as a valuable resource for a wide range of stakeholders, including policymakers, researchers, environmental organizations, and the general public by providing insights into pellet producers, production figures, fuel quality, market trends, environmental impacts, and other relevant information. This database would not only promote transparency but also stimulate informed decision-making and foster a more inclusive dialogue about the future of the pellet industry in Poland.

## Theory

The quality of the pellets plays a pivotal role in achieving efficient and reliable energy production and reducing the emission impact on human health and the environment. Therefore, comprehensive testing of solid biomass fuels is imperative to ensure their high quality, a crucial step in advancing the widespread adoption of sustainable and efficient biomass energy solutions. However, a significant number of biomass pellets on the Polish market are not certified, and limited information regarding fuel properties and biomass origin is available to consumers (Drobniak et al. 2022). The lack of mandatory and rigorous quality control and certification processes has opened the door to subpar products that not only compromise the efficiency of biomass heating systems but also raise environmental and safety concerns (Jelonek et al. 2020, 2021; Drobniak et al. 2021, 2023).

Beyond the quantity of the pellets tested, a noteworthy concern pertains to the type and frequency of fuel testing. Presently, standard testing procedures, such as ENplus and DINplus, follow a defined set and thresholds of physical and chemical parameters (DINplus 2023a; ENplus 2023). However, research suggests that the currently performed analyses may not suffice to ensure high quality of pellets available on the market, indicating the necessity for additional testing measures as, for example, highlighted in studies by Drobniak et al. (2021, 2022, 2023) and Jelonek et al. (2020, 2021). As for regularity of testing, following ENplus certification requirements, pellet plants get audited once a year to inspect the production process and the fuel quality management, with one additional unannounced annual collection of samples and testing of pellets and added inspections in case of pellet quality complaints (ENplus 2023).

Considering the urgent need to transition to sustainable and environmentally friendly energy sources, our study analyzes 30 biomass pellet samples available for retail purchase to customers in the Silesian region of south-central Poland. Our goal was to reanalyze these fuels and compare the results to the quality data provided by the pellet producers and the certification requirements. This approach allowed us to critically evaluate the quality of biomass fuels on the market and assess the accuracy and reliability of information disseminated by the fuel manufacturers. Ultimately, our research highlights the significance of adhering to certification requirements and the need to enhance the quality assessment of biomass pellets. By addressing these critical aspects, our research seeks to contribute to the broader understanding of the bioenergy industry in Poland, while also informing consumers, policymakers, and stakeholders about the quality of biomass pellets available for purchase.

## Methods

A total of 30 biomass pellet samples, each weighing 15 kg, were purchased from various retail outlets in the Upper Silesia region, located in the south-central part of Poland. These pellets were randomly acquired from home and garden stores, such as Castorama and Leroy Merlin, as well as popular in Poland fuel depots. Our objectives were to 1) evaluate the labeling of the fuel bags (information provided by the producer) and 2) to conduct a comprehensive evaluation of pellet quality in comparison to existing norms and certification requirements of DINplus and ENplus (wood pellets class A1 and non-wood pellets class A), as well as PL-US BIO that combines quality assessment following DINplus and optical microscopy analysis (PL-US BIO 2023).

All the samples underwent a rigorous physicochemical analysis, conducted both by an internal and an independent third-party accredited laboratory. This analysis was executed according to the DINplus certification standard (DINplus 2023b), ensuring a thorough examination of key physical and chemical attributes. These parameters included pellet diameter and length, fines content, mechanical durability, and bulk density, as well as moisture, ash, sulfur, and nitrogen content; ash melting temperatures; and net calorific value.

Additionally, samples underwent petrographic analysis to assess the composition of the pellets and to identify any potential contaminants. The preparation of the microscopic plugs, 1000-point petrographic analysis in reflected light along with the identification and classification of the pellet components was conducted following the methodology described by Drobniak et al. (2022, 2023). The identified components included woody and non-woody biomass, bark, charcoal, fossil and processed organic matter, inorganic matter, petroleum products, and binders and additives. Based on the data, the total amount of impurities in each sample was calculated.

## Results and discussion

### Fuel bag labeling

Transparent and accurate biomass pellet bag labeling provides essential information regarding fuel origin, composition, quality, and certifications. This information promotes consumers’ awareness and trust in making informed choices and confidence in the product’s quality. Transparent labeling also holds manufacturers accountable for the accuracy of their product information which, in turn, encourages responsible production practices and discourages deceptive marketing.

Nowadays, the market offers a wide variety of biomass fuel labeling designs, often with insufficient information or absent details regarding the biomass source and fuel characteristics. This situation can lead to confusion among consumers, who find it challenging to make informed product comparisons due to the lack of relevant information. Although ENplus, a leading certification scheme for wood pellets, has provided some guidelines for bag design (ENplus 2018), addressing this challenge requires a broader initiative and widespread standardization for consistent bag labeling and informative shopping experience.

**Fig. 2:**
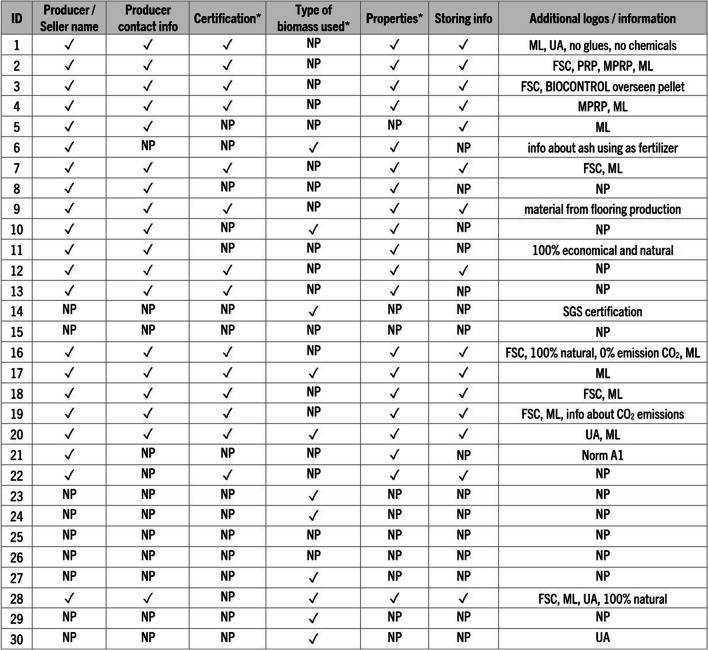
Information about the fuel provided by the producer on the purchased bags: ✓ – information was provided, NP – information not provided, FSC – fuel with responsible sourcing certificate, ML – bag with multilingual labeling, MPRP – producer is a member of Polish Pellet Council, PRP – product recommended by Polish Pellet Council, UA – pellet produced in Ukraine. * For a full list of the producers’ provided information, see Figs. 5, 6, 7, 8, 9, and 10 or data repository at 10.5281/zenodo.10843482

The biomass pellet market in Upper Silesia offers a diverse variety of bag designs. They range from minimalistic plastic bags or cardboard boxes with no product information to bags with elaborate designs containing fuel properties and eco-friendly graphics. While some bags featured details about biomass origin (typically very general), material properties, and quality certification logos, others provided very limited or no data (Fig. 2). Specifically, out of 30 pellets:

- 9 bags had no producer name (Fig. 3)
- 12 bags had no producer contact information
- 10 bags contained no fuel properties information
- 15 bags contained no fuel storage information
- 19 samples had no information about the type of biomass used to produce the fuel

**Fig. 3:**
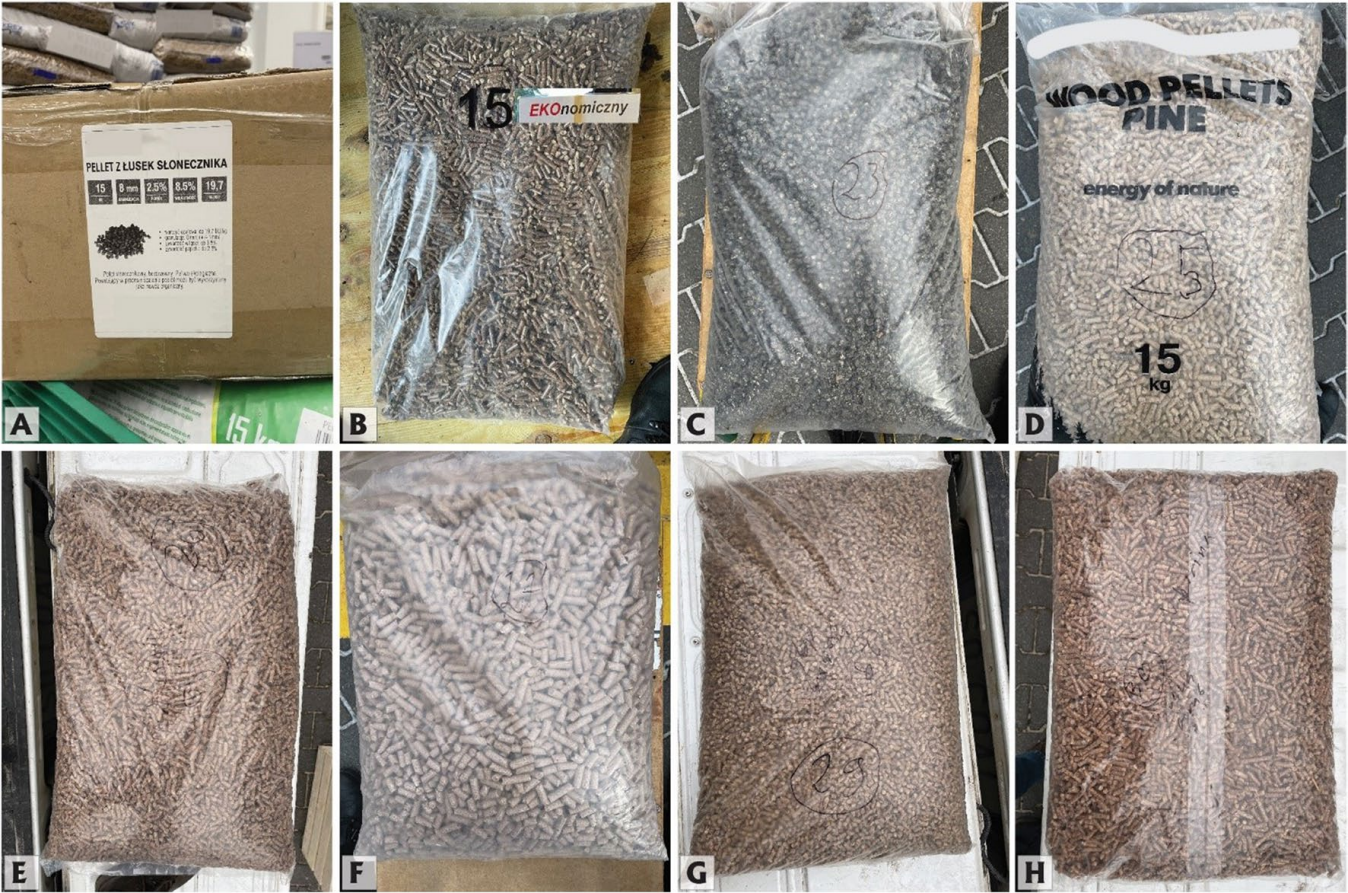
Examples of purchased biomass pellets with no or limited labeling on the bag and box: **A** – sample 6 (name of the producer was obscured), **B** – sample 15, **C** – sample 23, **D** – sample 25 (name of the producer was obscured), **E** – sample 26, **F** – sample 27, **G** – sample 29, **H** – sample 30

Producers provided also, sometimes in multiple languages, information regarding the fuel origin and certifications received:

- 14 bags displayed a DINplus or ENplus certification logo
- 6 bags contained an FSC (Forest Stewardship Council) logo—responsible sourcing certificate
- 2 bags displayed logos of the Polish Pellet Council
- 1 bag contained information of being BIOCONTROL overseen pellet
- 1 bag displayed a logo of SGS (Société Générale de Surveillance SA.) certification
- 4 purchased bags contained pellets produced in Ukraine

Furthermore, some of the bags contained additional information such as:

- material source (for instance from flooring production)
- statements that the product contains no glues or chemical additions
- the possibility of using ash as fertilizer
- the fuel being 100% economical and natural
- information that pellet production followed norm A1
- information about CO_2_ emissions:
- a) 0% emissions of CO_2_ (sample 16)
- b) CO_2_ emissions when the product is burned are equal to the amount of CO_2_ absorbed by the plant during growth, which means that no additional CO_2_ is emitted into the atmosphere (sample 19)

Some of the pellet bags were additionally marked with symbols and statements showcasing their high quality, high calorific value, good price, producer participation in a tree planting program (sample 9), the fuel being a biofuel of the future (sample 17), a source of positive energy (sample 22), or energy of the nature (sample 25). Many of the bags contained information about the possibility of recycling the bag.

### Physical properties of pellets

**Fig. 4:**
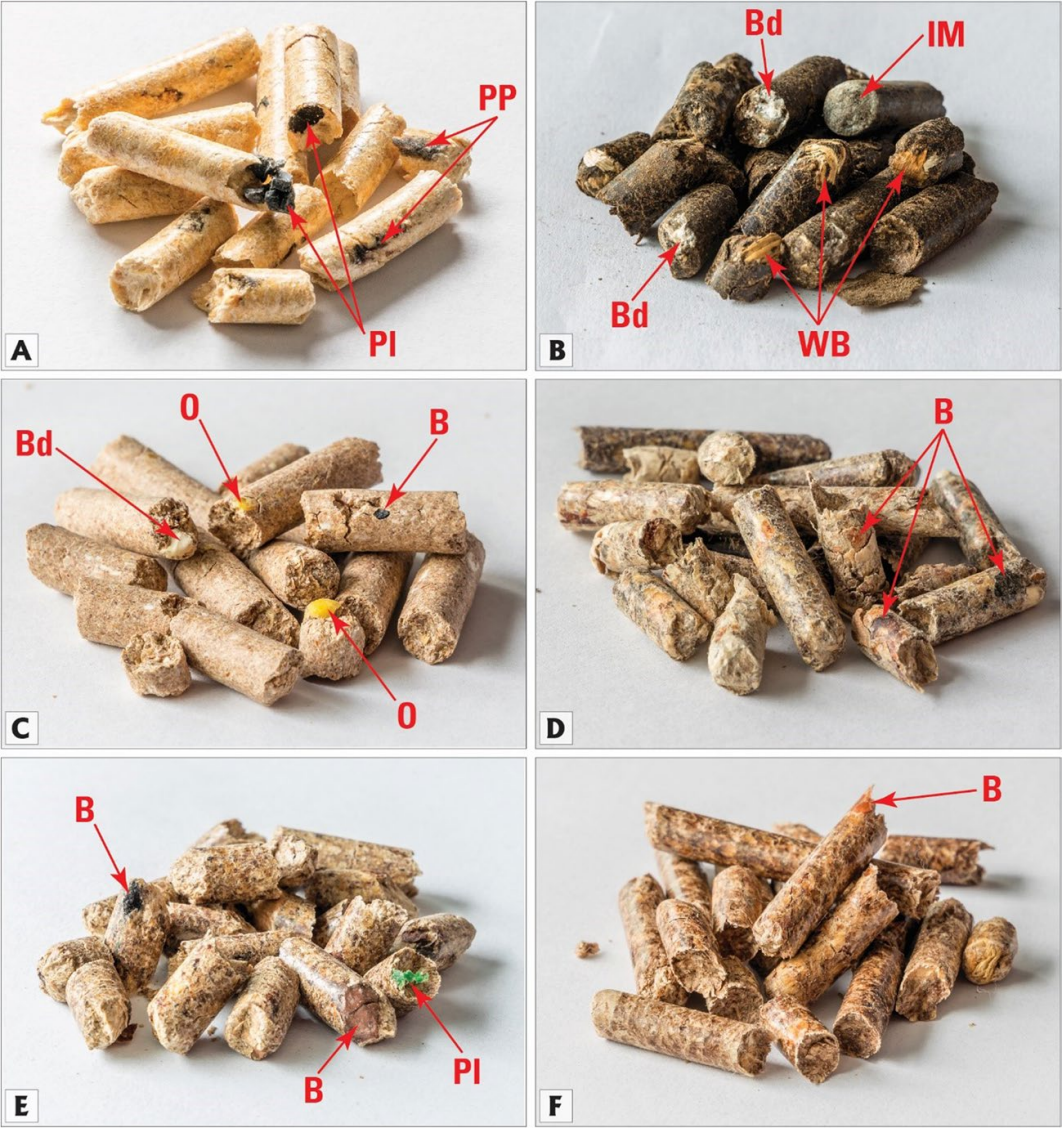
Examples of purchased biomass pellets: **A** – sample 6 – wood pellets with plastic (Pl) fragments and unidentified petroleum product (PP); **B** – sample 24 – sunflower husk and bean pods pellets with large pieces of woody biomass (WB), flour binder (Bd) and inorganic matter (IM); **C** – sample 27 – bran pellets with a fragment of bark (B), flour binder (Bd) and pieces of grains (O); **D** – sample 28 – coniferous wood pellets with pieces of bark (B); **E** – sample 29 – pine and oak wood pellets with a fragment of plastic (Pl) and large pieces of bark (B); **F** – sample 30 – pine and hornbeam wood pellets with a piece of bark (B). Component classification after Drobniak et al. (2023)

Purchased biomass pellets came in small cylindrical shapes and a variety of colors depending on the origin of the source materials and technology of production. Several of the samples exhibited an easily noticeable lack of material homogeneity, and some contained an assortment of impurities visible even to the naked eye (Fig. 4). The most typical undesired addition detected megascopically was bark, followed by pieces of plastic, inorganic matter, and clusters of flour binder.

**Fig. 5:**
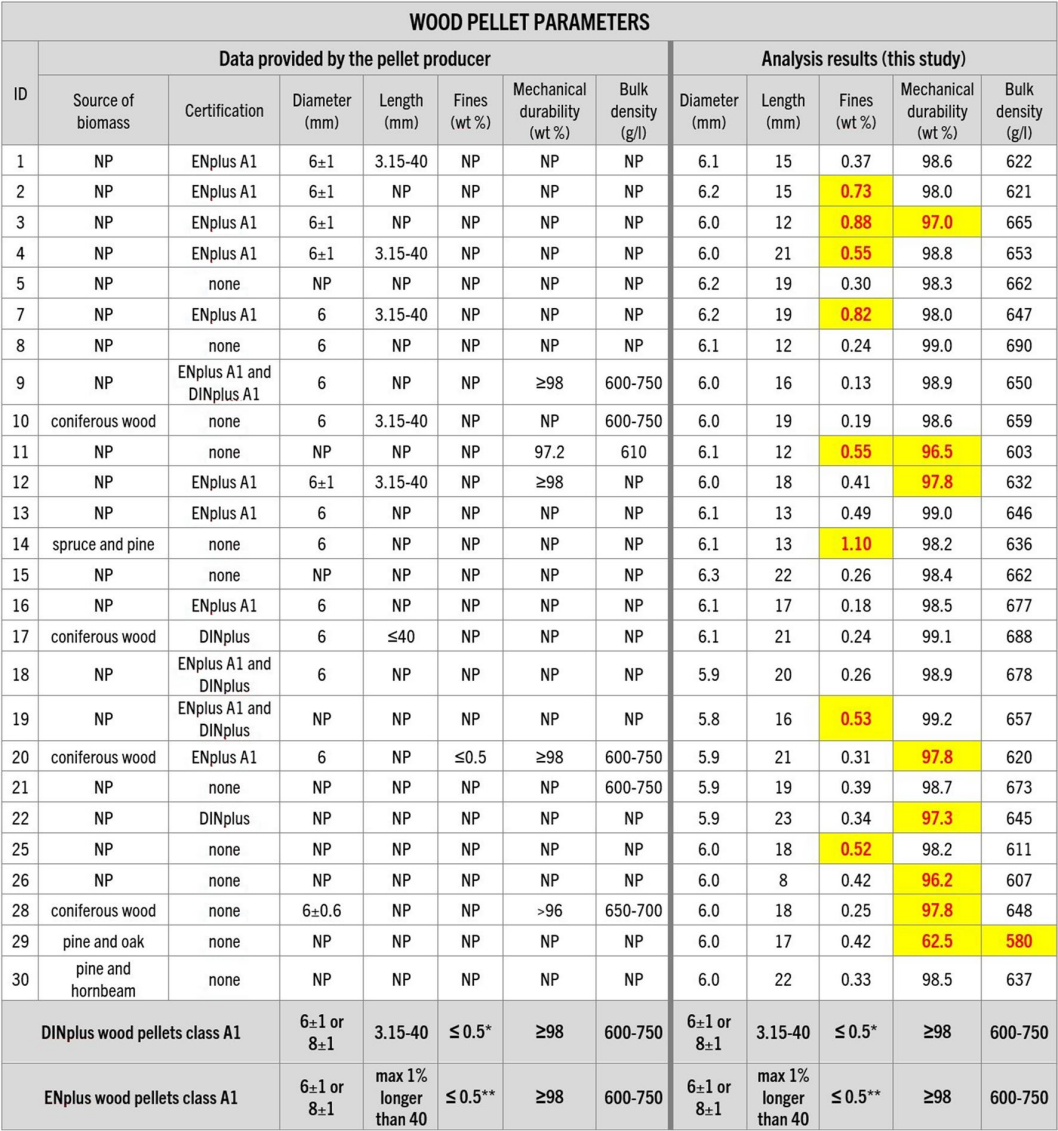
Information about the wood pellet parameters provided by their producers and analysis results regarding pellet diameter and length, fines, mechanical durability, and bulk density conducted in this study. Guidelines of DINplus and ENplus A1 certification requirements (in accordance with ISO 17225-2:2021) for wood were added for comparison (DINplus 2021; ENplus 2023). NP – data not provided, * – for bags up to 20kg, ** – at the factory gate or bag filling. Results that would not meet or are not meeting the certification criteria are highlighted

**Fig. 6:**
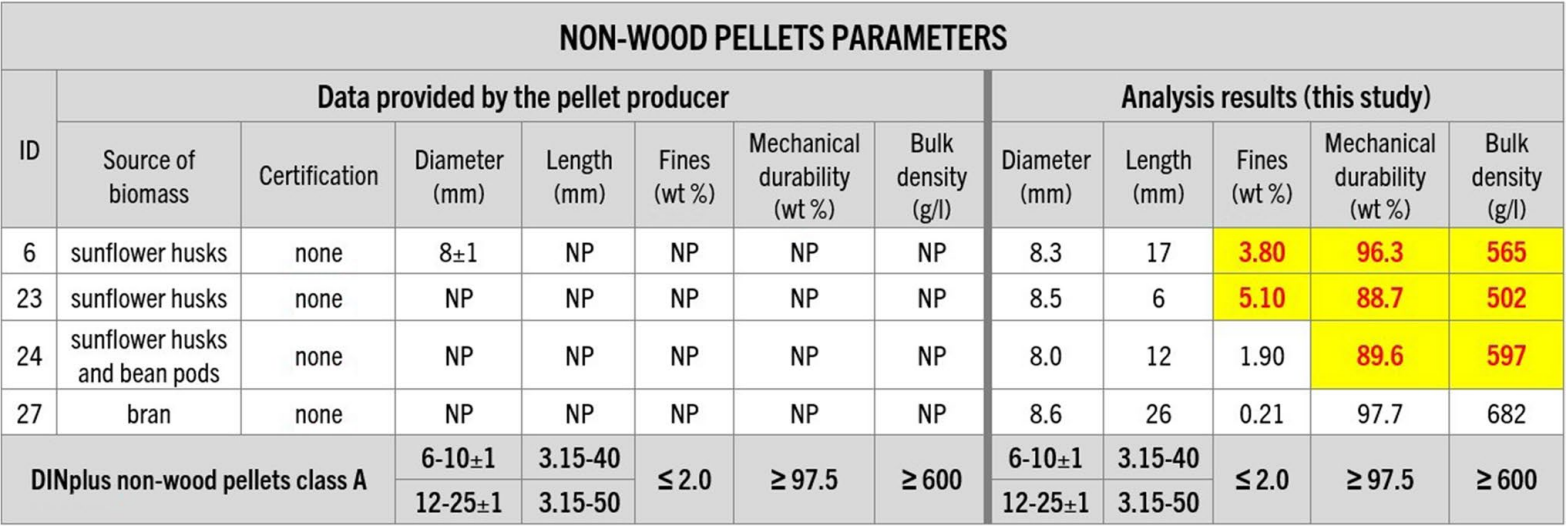
Information about the non-wood pellet parameters provided by their producers and analysis results regarding pellet diameter and length, fines, mechanical durability, and bulk density conducted in this study. Guidelines of DINplus certification requirements (in accordance with ISO 17225–6:2021) for non-wood pellets were added for comparison (DINplus 2023b). Results that would not meet the certification criteria are highlighted

Physical analyses of the samples (diameter and length, fines content, mechanical durability, and bulk density) revealed that the properties of some of the pellets are not within the DINplus and ENplus certification limits (even if the pellet was certified) and, in some cases, those properties do not correspond with parameter values provided by the pellet producer (Figs. 5 and 6).

While all purchased pellets met the diameter and length limits, values of mechanical durability and the content of fines were not within the certification thresholds for 10 out of the 30 samples. Additionally, three out of four non-woody pellets and one of the wood pellet samples (number 29) showed too low values of bulk density (Figs. 5 and 6). This shows that a significant number of purchased fuels are not strong enough to withstand mechanical stress during their production, handling, and transportation, which makes them prone to breaking apart and creating dust. This, in turn, affects the fuel transport and efficiency of combustion and may lead to boiler clogging, increased emissions of pollutants, and increased risk of fire or dust explosion (Williams et al. 2018; Gilvari et al. 2019, 2021; Kuranc et al. 2020).

### Chemical properties of pellets

**Fig. 7:**
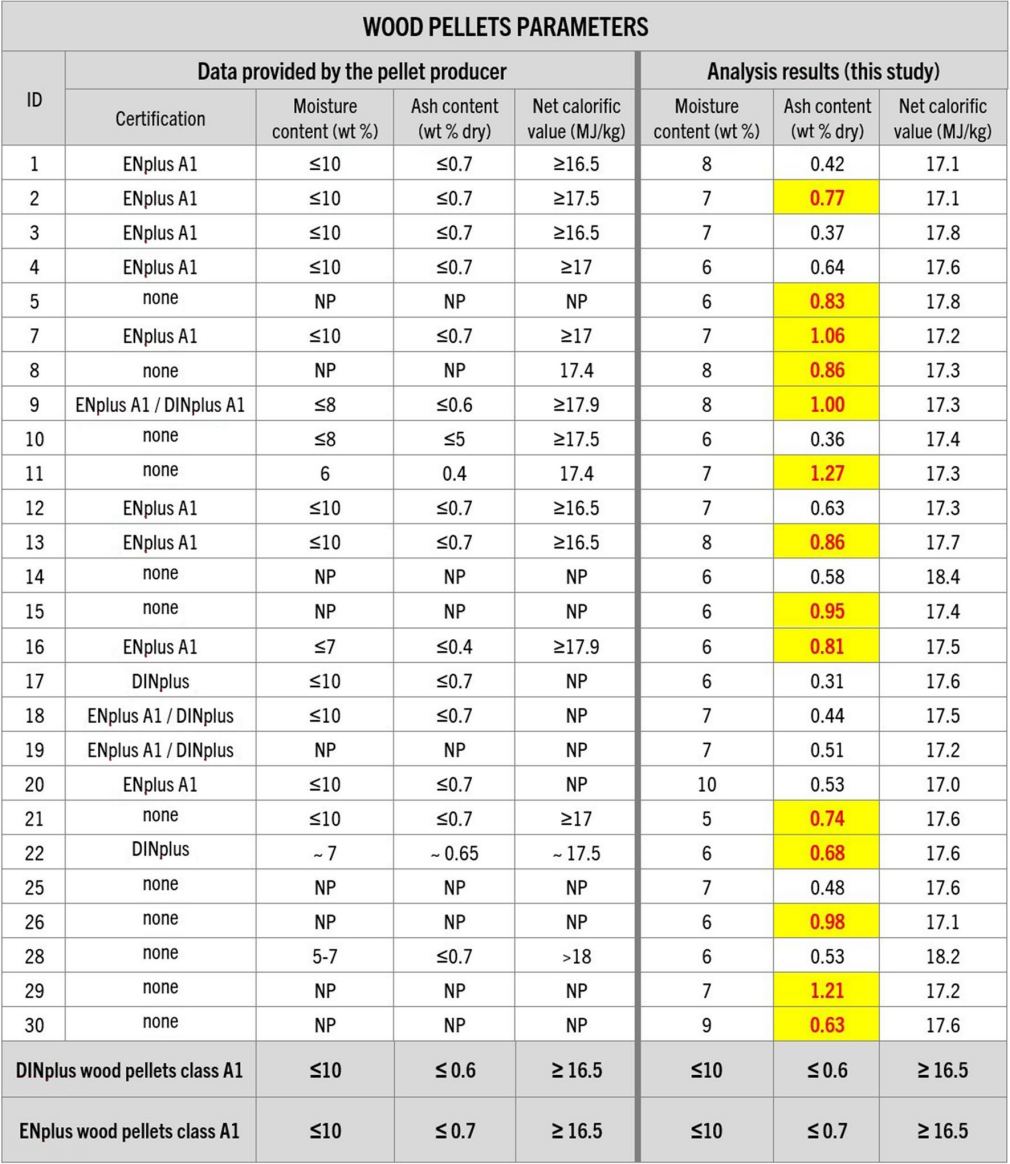
Information about wood pellet parameters provided by their producers and analysis results regarding pellet moisture, ash content, and the net calorific value conducted in this study. Guidelines of DINplus and ENplus A1 certifications (in accordance with ISO 17225-2:2021) for wood pellets were added for comparison (DINplus 2021; ENplus 2023). NP – data not provided. Results that would not meet or are not meeting the certification criteria are highlighted

**Fig. 8:**
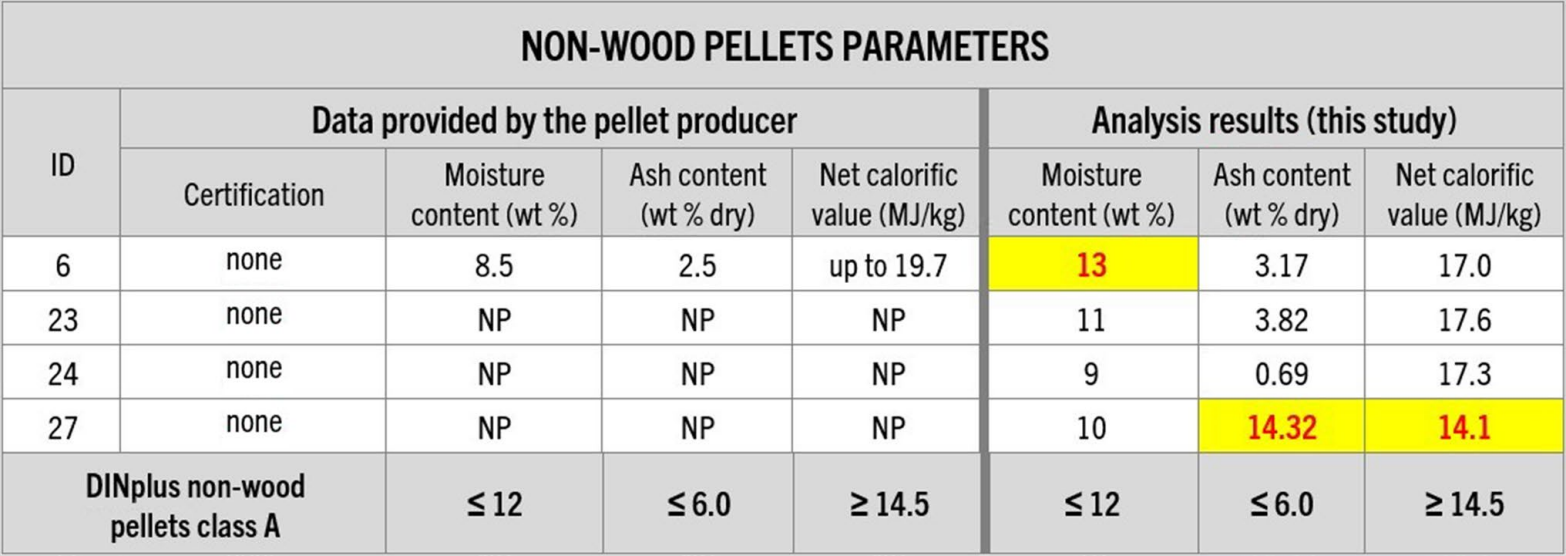
Information about the non-wood pellet parameters provided by their producers and analysis results regarding pellet moisture, ash content, and the net calorific value conducted in this study. Guidelines of DINplus certification (in accordance with ISO 17225–6:2021) for non-wood pellets were added for comparison (DINplus 2023b). NP – data not provided. Results that would not meet the certification criteria are highlighted

Chemical analysis of the purchased samples (moisture, ash, sulfur, and nitrogen content, ash melting temperatures, and net calorific value) showed that almost all of the samples had moisture content within the recommended range of up to 10 wt %. However, the majority (over 50%) of the fuels had ash content higher than the certification threshold (Figs. 7 and 8), with two of the samples (11 and 29) more than double the ash limit for DINplus certification. However, sample 27 (Fig. 8) yielded the highest amount of ash of all the samples, reaching 14.3 wt %, equivalent to 139% over the recommended limit, significantly impacting the fuel calorific value.

**Fig. 9:**
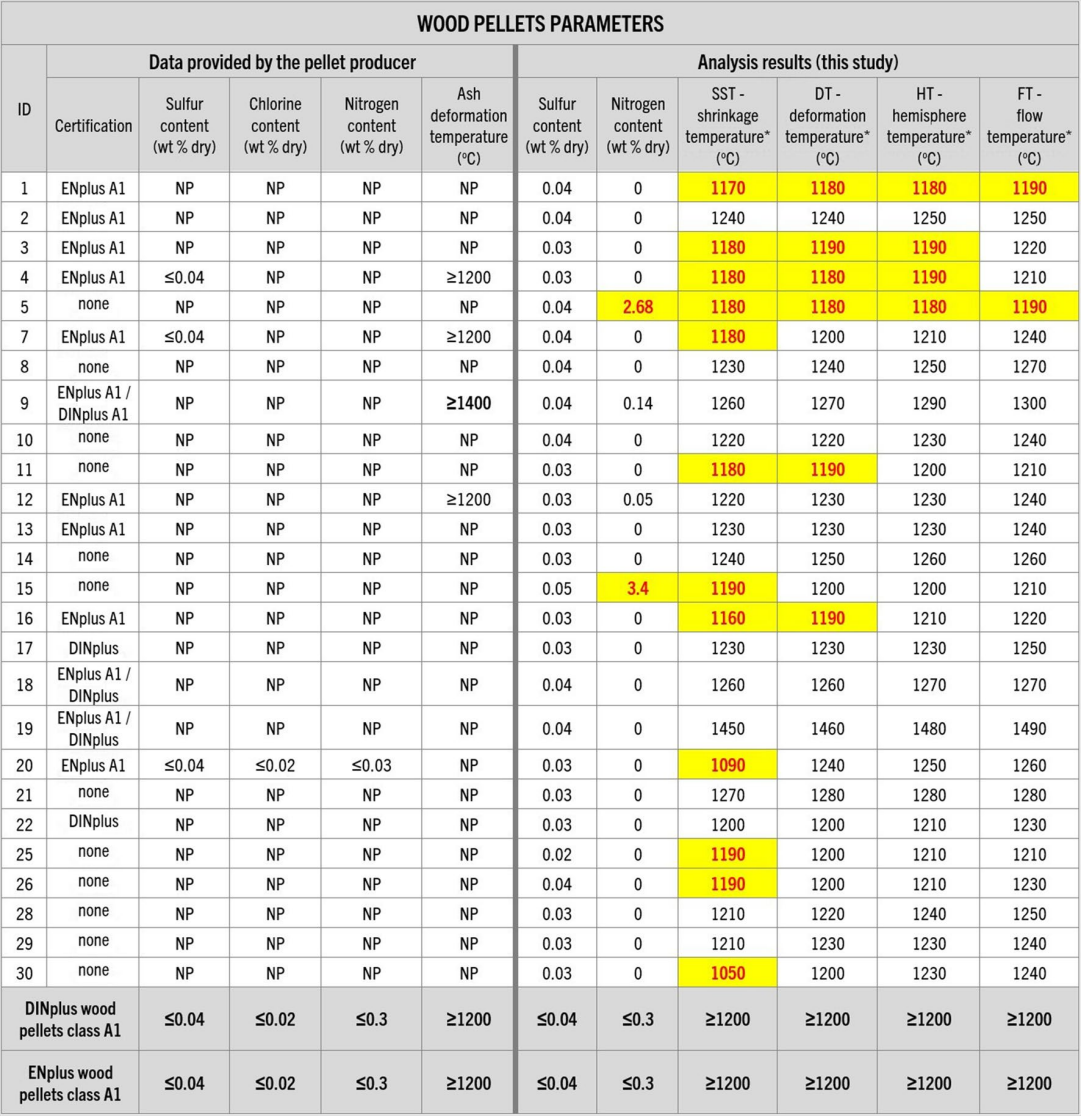
Information about the wood pellet parameters provided by their producers and analysis results regarding pellet sulfur, chlorine, and nitrogen content and ash deformation temperatures conducted in this study. Guidelines of DINplus and ENplus A1 certifications (in accordance with ISO 17225–2:^2021^) for wood pellets were added for comparison (DINplus 2021; ENplus 2023). NP – data not provided; * – oxidized conditions. Results that would not meet or are not meeting the certifications criteria are highlighted

**Fig. 10:**
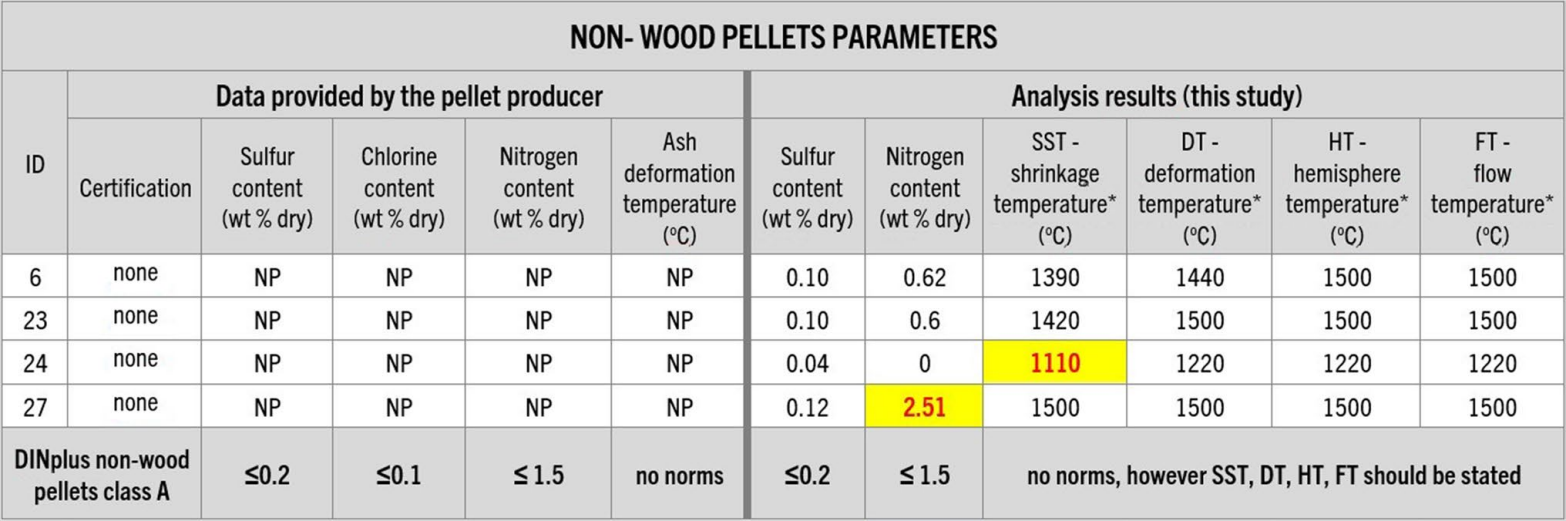
Information about the wood pellet parameters provided by their producers and analysis results regarding pellet sulfur, chlorine, and nitrogen content and ash deformation temperatures conducted in this study. Guidelines of DINplus certification (in accordance with ISO 17225–6:^2021^) for non-wood pellets were added for comparison (DINplus 2023b). NP – data not provided; * – oxidized conditions. Results that would not meet the certification criteria are highlighted

Another observed discrepancy between values provided by the pellet producers and data acquired in this study turned out to be ash melting temperature, which is closely related to boiler performance, combustion efficiency, and creation of sinter and slag deposits, which, in some cases can lead to boiler damage (Holubcik et al. 2015; Radačovská et al. 2017; Čajová Kantová et al. 2023). Almost half of the tested samples did not meet the certification guidelines of ENplus and DINplus which recommend an ash melting temperature above 1200 °C. These fuels (Figs. 9 and 10) showed a temperature lower than the certification thresholds by 10 to 150 °C. While norms do not exist for non-woody biomass regarding this parameter, sample 24 showed ash shrinking temperature below 1200 °C.

Three samples (5, 15, and 27) showed also significantly higher nitrogen content than DINplus and ENplus limits (Figs. 9 and 10). While the source of nitrogen in the pellets is unknown, nitrogen content might have implications for combustion efficiency, increased corrosion, fouling of a boiler, or formation of NO_x_ and ammonia, and therefore can lead to negative environmental impacts (Glarborg et al. 2003; Klason and Bai 2007; Petrocelli and Lezzi 2014; Schmid et al. 2020).

### Petrographic composition of pellets

Although numerous countries around the world have adopted quality standards to govern the quality of biomass pellets, several studies have identified challenges and issues with the current standardized testing and have advocated for reevaluation of the standards (Chandrasekaran et al. 2012; Duca et al. 2014; Rahman and Hopke 2017; Thiffault et al. 2019; Jelonek et al. 2020, 2021; Drobniak et al. 2021, 2022, 2023; Mencarelli et al. 2023). One proposed enhancement to the current testing methods is the adoption of petrographic analysis in reflected light (Drobniak et al. 2022, 2023). While this method is firmly established and routinely employed in the examination of coal, source rocks, metals, ceramics, and polymers, utilizing it for evaluating solid biomass fuels has been very limited. A recent development is the introduction of a groundbreaking PL-US BIO certification program in Poland that combines traditional physicochemical testing with reflected light microscopy analysis (PL-US BIO 2023).

**Fig. 11:**
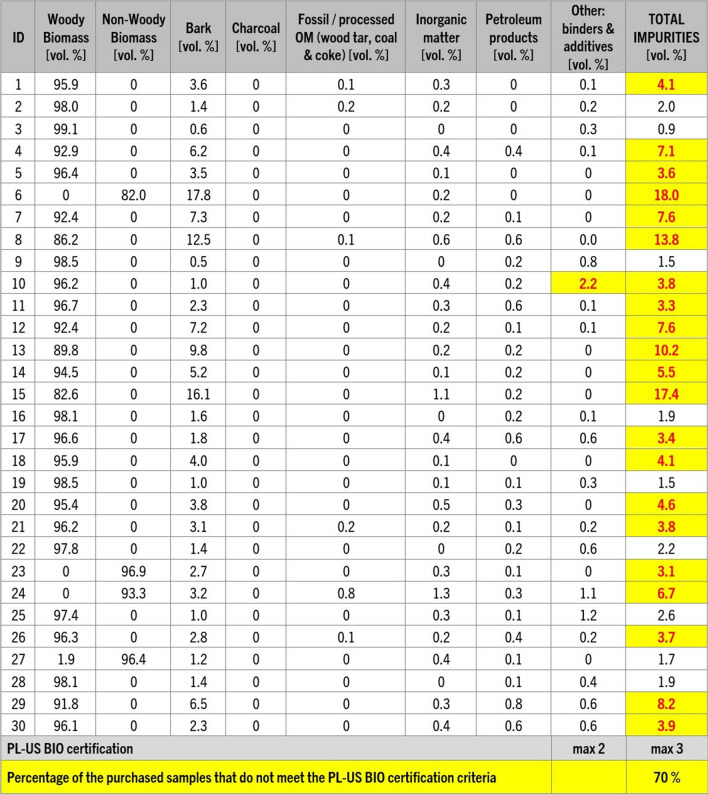
Results of petrographic analysis following the classification of solid biomass components by Drobniak et al. (2023). Results that would not meet the PL-US BIO certification (PL-US BIO 2023) criteria are highlighted

**Fig. 12:**
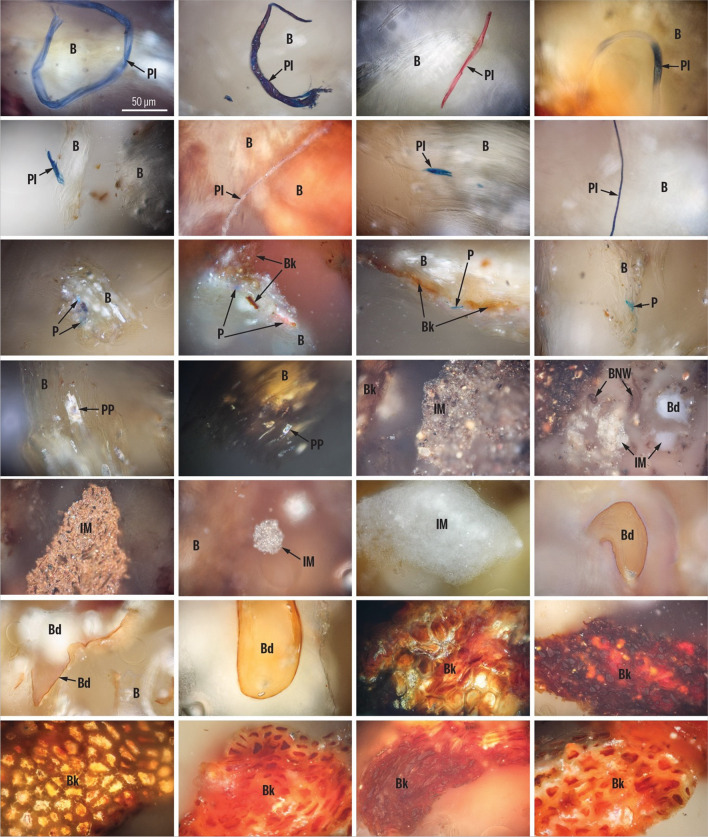
Photomicrographs showing examples of primary organic matter (B – biomass, BNW – non-woody biomass) and undesired additions (Bd – binder, Bk – bark, IM – inorganic matter, P – paint, Pl – plastic, PP – petroleum product (probably glue)) found in purchased pellets. Reflected white light, oil immersion, the scale bar is identical for all microphotographs. Classification of the components after Drobniak et al. (2023)

For the pellets studied, results obtained from petrographic analysis showed that 70% of the examined pellets would not meet the criteria set for PL-US BIO certification (PL-US BIO 2023) due to the presence of elevated levels of impurities. While nine of the 30 evaluated samples had impurities lower than the recommended 3 vol. %, for 21 samples, the level of undesired additions ranged volumetrically between 3.1 and 18% (Fig. 11). Among these undesirable inclusions, bark, present in quantities from 0.5 to 17.8 vol. %, emerged as the most frequent unwanted impurity (Figs. 12). While bark is a type of biomass, it has been considered an unwanted addition in pellet fuels due to its association with harmful particulate matter and smog emissions (Drobniak et al. 2022; Sippula et al. 2007). The presence of bark can also introduce an increased concentration of inorganic elements, including soil or sand residues that might originate from transportation. As a direct consequence, the ash content in pellets tends to be higher when bark is a significant component. Furthermore, when bark content surpasses a threshold of approximately 2.5% to 3%, it has been observed to correlate with the formation of sinter and slag during combustion (Holubcik et al. 2015; Radačovská et al. 2017; Drobniak et al. 2022; Čajová Kantová et al. 2023).

Other impurities found in the pellets included inorganic matter (0.1 to 1.3 vol. %); traces of petroleum products, most commonly glues and plastic (0.1 to 0.8 vol. %); and fossil fuels (up to 0.8 vol. %). The content of binders (typically flour) and additives was estimated from 0 to 2.2%, with one sample (number 10) containing a binder content level above the certification threshold.

## Summary and conclusions

Biomass pellets have been gaining significant importance as a sustainable and renewable source of energy. As a result, the biomass industry in Poland has been experiencing substantial growth driven by the country’s need for energy independence and demand for energy from renewable sources. However, to attain renewable energy objectives, optimize utilization efficiency, and mitigate environmental and human health impacts, efforts to produce pellets of consistently high quality are imperative.

The quality of biomass pellets is of paramount importance for the efficiency and environmental sustainability of biomass energy production. Yet, our assessment of the fuels randomly purchased in Upper Silesia’s stores revealed that merely 14 out of the 30 acquired pellet fuels possessed ENplus and/or DINplus certifications and many of the samples fall outside the established certification thresholds. This low certification rate and significant quality variation in the characteristics of pellets highlight the absence of a uniform, nationwide quality control system, resulting in the presence of numerous untested and low-quality fuels on the market.

When it comes to the purchased 30 Polish pellets, as indicated in Figs. 2 and 3, significant enhancement in bag labeling is a compelling necessity. Our findings reveal that 30% of the purchased bags lacked the producer’s name, 40% omitted any form of producer contact information, 63% failed to disclose information about the type of biomass used, 33% did not provide any details regarding pellet properties, and 50% did not offer fuel storage instructions. The absence of such crucial information emphasizes the urgency for the establishment of national guidelines in biomass pellet labeling and oversight of labeling practices. Additionally, due to the scarcity of information, the creation of a comprehensive and publicly accessible Polish national database encompassing solid biomass fuel producers and manufactured fuels would be of great value. Such a database would serve as an invaluable reference point for anyone seeking to gain insights into the origin and characteristics of the pellets available for purchase. Moreover, it would represent an essential resource for the compilation of biomass industry statistics for researchers, policymakers, and the public.

**Fig. 13:**
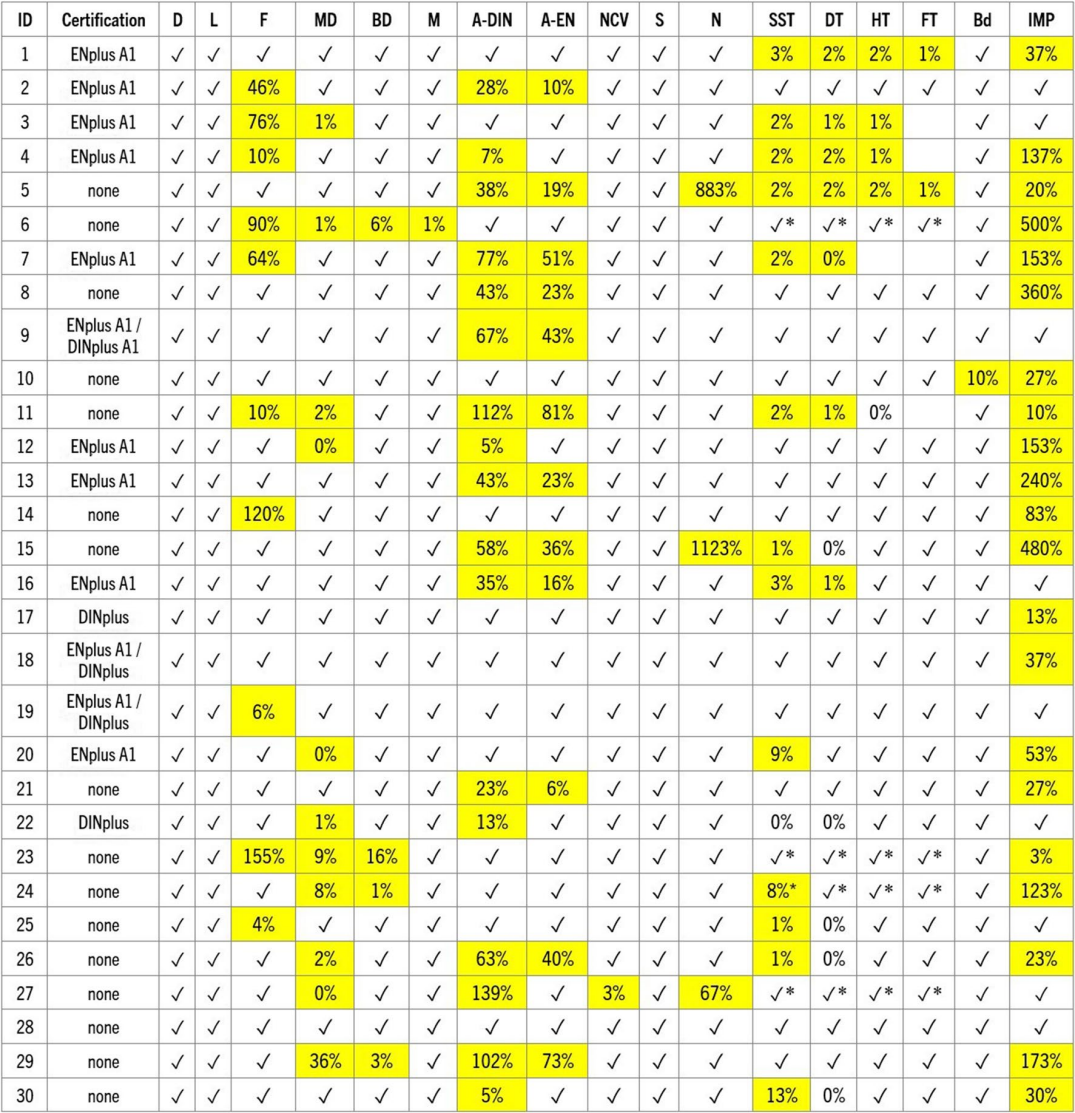
Range of differences in percentage between the results of the study and the certification limits. D – pellet diameter, L – pellet length, F – fines content, MD – mechanical durability, BD – bulk density, M – moisture content, A-DIN – ash content following DINplus certification A1, A-EN – ash content following ENplus certification A1, NCV – net calorific value, S – sulfur content, N – nitrogen content, SST – shrinkage temperature, DT – deformation temperature, HT – hemisphere temperature, FT – flow temperature, Bd – binder content following the PL-US BIO certification, IMP – total amount of impurities following the PL-US BIO certification, ✓ – value within the DINplus and/or ENplus certification limits, * – no norm exists, DINplus levels added for comparison. Results that would not meet the certification criteria are highlighted

Testing revealed considerable discrepancies between the producer-provided information and analytical results of this study, particularly concerning mechanical durability, and ash and fines content (Figs. 5, 6, 7, 8, 9, and 10). In many cases, even samples with certificates did not meet the established quality standards limits. While this difference was relatively minor for some pellets, for others, it significantly exceeded certification threshold values (Fig. 13). It was observed that 10 of the samples exceeded the fines content certification limit by a considerable margin, ranging from 6 to a staggering 155% over the specified threshold. This is indicative of a substantial deviation from the quality standards, which can have far-reaching implications for the performance and efficiency of the pellets as a heating source.

The issue of increased ash content in the tested pellets was also prevalent, with a majority of the samples exceeding DINplus certification limits, maximum acceptable ash content of 0.6% for woody pellets class A1 and 6% for non-woody fuels class A. Nevertheless, more than half of the samples displayed ash content levels ranging from 5 to as much as 139% over the stipulated limits (Fig. 13). However, the most remarkable instance can be observed in the nitrogen content of samples 5 and 15, which surpassed the specified limit by an astonishing 883% and 1123%, respectively (Fig. 13). Moreover, 70% of the samples showed an elevated presence of unwanted additives like bark, mineral matter, and petroleum-based products. Petrographic analysis revealed that impurities values were exceeded by 3% to 500%. This situation is particularly disconcerting given that some of the pellets had received ENplus and/or DINplus certification, which should ensure their high quality (Figs. 11 and 13).

The results of our study show that among all 30 purchased samples, only one would pass the quality thresholds set by PL-US BIO certification. This result indicates the necessity for enhanced focus on the pellet manufacturing process and the selection of raw materials, as fuel parameters exceeding the standards can compromise combustion efficiency, escalate boiler maintenance requirements, and potentially give rise to environmental issues due to elevated emissions. In light of this assessment, it becomes imperative to enhance fuel quality assessment, incorporating advanced techniques like optical microscopy in addition to conventional standard testing procedures. To achieve that change, national regulatory measures would have to be implemented.

However, we would like to stress that the 30 biomass pellet samples studied may not be representative of the biomass pellets available on the Polish market, and therefore, the conclusions should not be generalized. Nevertheless, the results clearly emphasize an important issue of pellet quality and indicate weaknesses of the current certification schemes and shortcomings of the data provided to customers.

The low quality of a significant number of biomass pellets on the Polish market gives rise to concerns that can affect a broad spectrum of bioenergy aspects including not only energy efficiency but also environmental sustainability, public health, and the long-term viability of biomass as a renewable and clean energy source. This is especially critical as 2022 pellet production data are placing Poland as the sixth biggest pellet producer in the world, and Poland has also emerged as a significant pellet exporter. The research highlights the necessity for enhanced fuel quality control protocols in Poland, transparent and precise product labeling, and the establishment of a comprehensive national database of biomass fuel producers and fuels sold. These fundamental measures are crucial in raising customer awareness and fostering trust, ultimately promoting the broader adoption of biomass fuels as a dependable and sustainable energy source.

## Data Availability

The data that support the findings of this study are openly available in the Zenodo repository at 10.5281/zenodo.10843482.
